# Endogenous Type I-C CRISPR-Cas system of *Streptococcus equi* subsp. *zooepidemicus* promotes biofilm formation and pathogenicity

**DOI:** 10.3389/fmicb.2024.1417993

**Published:** 2024-05-22

**Authors:** Honglin Xie, Riteng Zhang, Ziyuan Li, Ruhai Guo, Junda Li, Qiang Fu, Xinglong Wang, Yefei Zhou

**Affiliations:** ^1^Department of Life Science, Nanjing Xiaozhuang University, Nanjing, China; ^2^College of Veterinary Medicine, Northwest A&F University, Yangling, China; ^3^School of Life Science and Engineering, Foshan University, Foshan, China

**Keywords:** *Streptococcus equi* subsp. *zooepidemicus*, Type I-C CRISPR-Cas system, biofilm, adhesion, RNA-Seq, virulence

## Abstract

*Streptococcus equi* subsp. *zooepidemicus* (SEZ) is a significant zoonotic pathogen that causes septicemia, meningitis, and mastitis in domestic animals. Recent reports have highlighted high-mortality outbreaks among swine in the United States. Traditionally recognized for its adaptive immune functions, the CRISPR-Cas system has also been implicated in gene regulation, bacterial pathophysiology, virulence, and evolution. The Type I-C CRISPR-Cas system, which is prevalent in SEZ isolates, appears to play a pivotal role in regulating the pathogenicity of SEZ. By constructing a Cas3 mutant strain (ΔCas3) and a CRISPR-deficient strain (ΔCRISPR), we demonstrated that this system significantly promotes biofilm formation and cell adhesion. However, the deficiency in the CRISPR-Cas system did not affect bacterial morphology or capsule production. *In vitro* studies showed that the CRISPR-Cas system enhances pro-inflammatory responses in RAW264.7 cells. The ΔCas3 and ΔCRISPR mutant strains exhibited reduced mortality rates in mice, accompanied by a decreased bacterial load in specific organs. RNA-seq analysis revealed distinct expression patterns in both mutant strains, with ΔCas3 displaying a broader range of differentially expressed genes, which accounted for over 70% of the differential genes observed in ΔCRISPR. These genes were predominantly linked to lipid metabolism, the ABC transport system, signal transduction, and quorum sensing. These findings enhance our understanding of the complex role of the CRISPR-Cas system in SEZ pathogenesis and provide valuable insights for developing innovative therapeutic strategies to combat infections.

## Introduction

*Streptococcus equi* subsp. *zooepidemicus* (SEZ) is a Lancefield group C beta-hemolytic *streptococcus*, known for causing various diseases in several animal species including horses, pigs, and dogs (Timoney et al., 1995; Walker and Runyan, 2003; Fan et al., 2008). SEZ is zoonotic, with transmission documented from animals such as horses, dogs, and guinea pigs to humans, often resulting in severe invasive diseases (Pelkonen et al., 2013). In the 1970s, a major SEZ epidemic in Sichuan, China resulted in over 300,000 pig fatalities (Ma et al., 2011, 2015). The pathogen isolated from this outbreak was identified as the ATCC35246 strain. By 2019, SEZ had emerged as a novel pathogen in North America, notably causing high mortality outbreaks in Ohio and Tennessee with death rates of 30 to 50% within two weeks post-infection (Chen et al., 2020). In 2021, Indiana reported an SEZ outbreak affecting 2-year-old adult sows, resulting in 66 deaths over a 6-week period, corresponding to a mortality rate of 2.75% (Chen et al., 2023).

The CRISPR-Cas (Clustered Regularly Interspaced Short Palindromic Repeats-CRISPR Associated) system, prevalent in most archaea and many bacteria, primarily serves as a defense mechanism against foreign nucleic acids from phages, plasmids, and viruses (Barrangou and Marraffini, 2014; Hille et al., 2018). The system is categorized into two main classes, which are further divided into six types and at least 33 subtypes (Makarova and Koonin, 2015). There is growing evidence that the CRISPR-Cas system also influences other bacterial biological processes such as antibiotic resistance, stress responses, virulence, and immune evasion (Sampson et al., 2013; Devi et al., 2022; Wang et al., 2022; Zakrzewska and Burmistrz, 2023; Xu et al., 2024). For instance, in *Francisella novicida*, the Type II-A CRISPR-Cas system downregulates bacterial lipoprotein (BLP) expression, thereby enhancing both pathogenesis and commensalism (Sampson et al., 2013). Similarly, in *Pseudomonas aeruginosa*, the Type I-F CRISPR-Cas system prevents biofilm formation through crRNA-guided targeting that damages integrated prophage DNA (Heussler et al., 2015). Additionally, studies on *Streptococcus agalactiae* have shown that CRISPR-mediated regulation of endogenous transcripts significantly reduces its ability to adhere, invade, and cause cytotoxic effects on host cells (Dong et al., 2021). Further research indicates that CRISPR inhibits the production of capsular polysaccharides through the suppression of *cpsA* promoter activity by CRISPR RNA (crRNA) (Nie et al., 2022).

Waller and Robinson (2013) reported that the Type I-C CRISPR-Cas system is widely present in SEZ strains, indicating its critical role in maintaining genomic integrity and responding to invasive genetic elements. However, the impact of this system on the pathogenicity of SEZ has not been documented. In this study, we constructed ΔCas3 and ΔCRISPR mutant strains and conducted transcriptomic profiling. Other than that, we utilized phenotypic methods, including biofilm assays, adhesion tests, cellular infection assays, and mice mortality studies, to investigate the effects of these mutations. These approaches confirmed the crucial role of the CRISPR-Cas system in enhancing virulence. Overall, these findings deepen our understanding of the complex role of the Type I-C CRISPR-Cas system in SEZ and provide new insights into its contribution to bacterial pathogenesis.

## Materials and methods

### Bacterial strains, plasmids, culture conditions

The bacterial strains and plasmids used in this study are detailed in Supplementary Table S2. The SEZ ATCC35246 bacterial strain was preserved in our laboratory for research purposes. SEZ strain were grown in Todd–Hewitt broth (THB) at 37°C. The RAW264.7 macrophages and PK-15 cells were also maintained in our laboratory. These cell lines were cultured in DMEM, supplemented with 10% fetal bovine serum (FBS), and maintained in a humidified 5% CO_2_ incubator at 37°C.

### Construction of the ΔCas3 and ΔCRISPR mutant strains

The deletion of the Cas3 or CRISPR in SEZ was conducted following a previously established protocol (Takamatsu et al., 2001). In sum, we initiated the process by using PCR amplification to generate upstream and downstream fragments of the target gene with specific primers (Supplementary Table S2). Then, these PCR products were ligated into the temperature-sensitive suicide vector, pSET-4s, using appropriate restriction enzymes. The resulting plasmids, were introduced into SEZ through electroporation.

Bacterial cultures were maintained at 28°C in the presence of 100 μg/mL spectinomycin (Spc). Upon reaching the mid-logarithmic growth phase, the bacteria were sub-cultured in THB supplemented with 100 μg/mL Spc and further incubated at 28°C until the early logarithmic phase was achieved. The culture was subsequently shifted to 37°C for a period of 4 h to facilitate plasmid loss. Cells were then plated on TSA plates and incubated at 28°C. Colonies that lost resistance to Spc at 37°C were selected, indicating the successful loss of the spectinomycin resistance gene carried by the plasmid. PCR analysis confirmed the creation of the double-crossover homologous recombination mutants, designated as ΔCas3 and ΔCRISPR.

### Cell adhesion

To investigate the impact of CRISPR-Cas system on SEZ adhesion to cells, we conducted the following procedure (Tang et al., 2017). SEZ strains were cultured, harvested, and washed with sterile PBS, then added to PK15 cells cultured in 12-well dishes at a multiplicity of infection (MOI) of 10. The cell-bacteria mixtures were co-incubated for 1 h. After incubation, non-adherent bacteria were removed by washing the cells with PBS. The cells were then lysed in sterile water, diluted appropriately, and plated on tryptic soy agar (TSA) plates for colony counting.

### Bacterial growth and hydrophobic assays

To assess the growth characteristics of SEZ strains, a single colony from each strain was cultured overnight at 37°C in THB medium. The cultures were then adjusted to an optical density at 600 nm of 0.3 using fresh THB medium. Subsequently, the cells were diluted at a 1:1,000 ratio into fresh THB medium and incubated at 37°C with shaking at 180 rpm for 15 h. Cell densities were measured at OD_600_ every hour.

To evaluate the hydrophobic properties of the bacteria, an equal volume of bacterial suspension was mixed with xylene for 2 min. The mixture was allowed to stand for 30 min to permit phase separation. The OD_600_ of the aqueous phase was then measured. The hydrophobicity percentage (H%) was calculated using the formula: H% = [(H_0_ − H)/H_0_] × 100%, where H_0_ is the initial absorbance before mixing with xylene, and H is the absorbance after phase separation (Del Re et al., 2000). In this assay, the SEZ capsule-deficient strain Δ*hasB* served as the control.

### Biofilm-formation assay

Biofilm formation was quantified by the crystal violet assay (Bonifait et al., 2008; Wang et al., 2011). In brief, 10 μL of the bacterial suspension was added to each well of 96-well flat-bottomed plastic culture plates containing 90 μL of THB supplemented with 10 mg/mL fibrinogen. The plates were incubated at 37°C for 24 h. After incubation, the culture medium was discarded, and the wells were gently washed three times with sterile PBS to remove non-adherent cells. The adherent bacteria were then fixed with methanol for 30 min and air-dried. A 1% crystal violet solution was applied to each well, and the plates were stained for 10 min at room temperature. Excess crystal violet was removed by rinsing the wells multiple times with distilled water. The biofilm-associated crystal violet was solubilized with 100 μL of 95% (v/v) ethanol, and the optical density at 570 nm was measured using a microplate reader.

For scanning electron microscopy (SEM) was performed as previously described (Xie et al., 2024), SEZ was inoculated into a 24-well culture plate containing sterile glass slides and incubated at 37°C for 48 h. The slides were then removed, washed three times with sterile PBS, and fixed in a solution of 5% glutaraldehyde, 4.4% formaldehyde, and 2.75% picric acid in 0.05% sodium cacodylate buffer for at least 1 h. The sample underwent sequential dehydration with 25, 50, 70, 80, and 95% ethanol.

The confocal laser scanning microscopy (CLSM), each biofilm was stained with 0.3% SYTO-9 and incubated in darkness for 15 min to allow for fluorescent labeling. The analysis of the biofilm images was conducted using Zeiss confocal software, enabling the acquisition of z-stack images.

### Transmission electron microscope

The bacterial suspension was prepared by centrifuging at 1,500 rpm for 10 min and then fixed in 2.5% glutaraldehyde for over 2 h. After fixation, the samples were dehydrated in propylene oxide for 10 min, embedded in epoxy resin, and shaped into trapezoids with a surface area. Ultramicrotomy was then used to cut the embedded samples into sections ranging from 50 to 90 nm in thickness. These thin sections were mounted onto 300 mesh copper grids, stained with alcoholic uranyl acetate and alkaline lead citrate, and then washed with distilled water. The prepared samples were observed using transmission electron microscope.

### RAW264.7 macrophages infection

RAW264.7 macrophages were cultured in 12-well plates without antibiotics. SEZ strains were added at a multiplicity of infection (MOI) of 1, and the mixture was co-incubated for 12 h. Quantitative real-time reverse-transcription polymerase chain reaction (qRT-PCR) was used to assess the transcription levels of pro-inflammatory cytokines (IL-1β, IL-6, IL-10, IL-18, TNF-α) and NF-κB. Briefly, post-infection, the cells were washed twice with sterile PBS. Total RNA was extracted using TRIzol reagent (Sigma, Shanghai, China), followed by cDNA synthesis using the Takara reverse transcription kit. Real-time quantitative PCR was performed using SYBR Green Premix (Takara, Beijing, China) on a LightCycler 480 fast real-time cycler. Fold changes were calculated using the 2^−ΔΔCt^. The data are presented as the means ± standard deviation (SD) of triplicate reactions for each gene transcript, and the primer sequences are provided in Supplementary Table S2.

### Mice survival curve and tissue bacterial load

Four-to-six-week-old BALB/c mice were obtained from Beijing Vetonlitech Experimental Animal Technology Co. Bacterial suspensions were prepared by diluting the bacteria in PBS, and 1 × 10^6^ CFU of SEZ strains were administered via intraperitoneal injection. Tissue bacterial load assays were conducted as previously described (Wei et al., 2012). Briefly, mice were euthanized 24 h after infection, and their spleens, livers, kidneys, and lungs were collected and weighed. Then, the tissues were homogenized and diluted in sterile saline (g/mL). The homogenized tissue samples were plated onto TSA plates for culturing, and the colonies were counted.

### Transcriptomic analysis

RNA preparation and RNA-seq analysis were performed as previously described (Xie et al., 2024). The ΔCas3, ΔCRISPR mutant, and wild-type strains were incubated in THB on a shaker at 37°C for 10 h to reach the logarithmic phase of growth. After incubation, the cells were harvested by centrifugation at 6,000 g for 10 min at 4°C. Total RNA samples were then extracted using the TRIzol method.

Library preparation followed a standardized protocol. Libraries meeting quality control criteria underwent PE150 sequencing on the Illumina platform. Raw sequencing data were processed into FASTQ files, including read sequences and corresponding quality scores. The data sets generated for this study can be found in the NCBI Sequence Read Archive (SRA) repository under BioProject accession number PRJNA1100188.

Before conducting the differential expression analysis, we conducted quality control on the sequencing reads and trimmed the adapter sequences. The sequencing reads from each library were then used for differential expression analysis with the read counts. As a reference genome sequence, we used the complete genome sequence of *Streptococcus equi* subsp. *zooepidemicus* (ATCC35246) and its genome annotation from the NCBI nucleotide database (accession number: NC_017582.1).

To compare gene expression in the ΔCas3 and ΔCRISPR mutants, genes with absolute log2 fold change values of ≥1 and false discovery rate (FDR) *p*-values of ≤0.05 were identified as differentially expressed (Supplementary Tables S3, S4). The functional annotations of these differentially expressed genes were performed using the Gene Ontology (GO) and KEGG databases. For further analysis of these genes, we utilized the STRING database, which provides functional enrichment analysis and explores protein–protein interaction networks using the STRING mapper tool (Szklarczyk et al., 2023).

### Data analysis

Statistical analyses were performed using one-way ANOVA with GraphPad Prism version 9.0 (GraphPad Software Inc., United States). Significant differences between groups are indicated by asterisks: ^*^p < 0.05, ^**^p < 0.01, and ^***^p < 0.001. Different letters denote significant differences within each strain, using a threshold of *p* < 0.05 for significance. All data are presented as means ± standard error of the mean (SEM), based on a minimum of three independent experiments.

## Results

### Characterization of the CRISPR-Cas system in SEZ

We collected whole-genome sequencing data of SEZ strains associated with high mortality from isolates in China (ATCC 35246 and CY strains) and isolates in the United States (2019s-2021s). The presence of the Type I-C CRISPR-Cas system was detected in all SEZ outbreak isolates (Supplementary Table S1).

**Figure 1:**
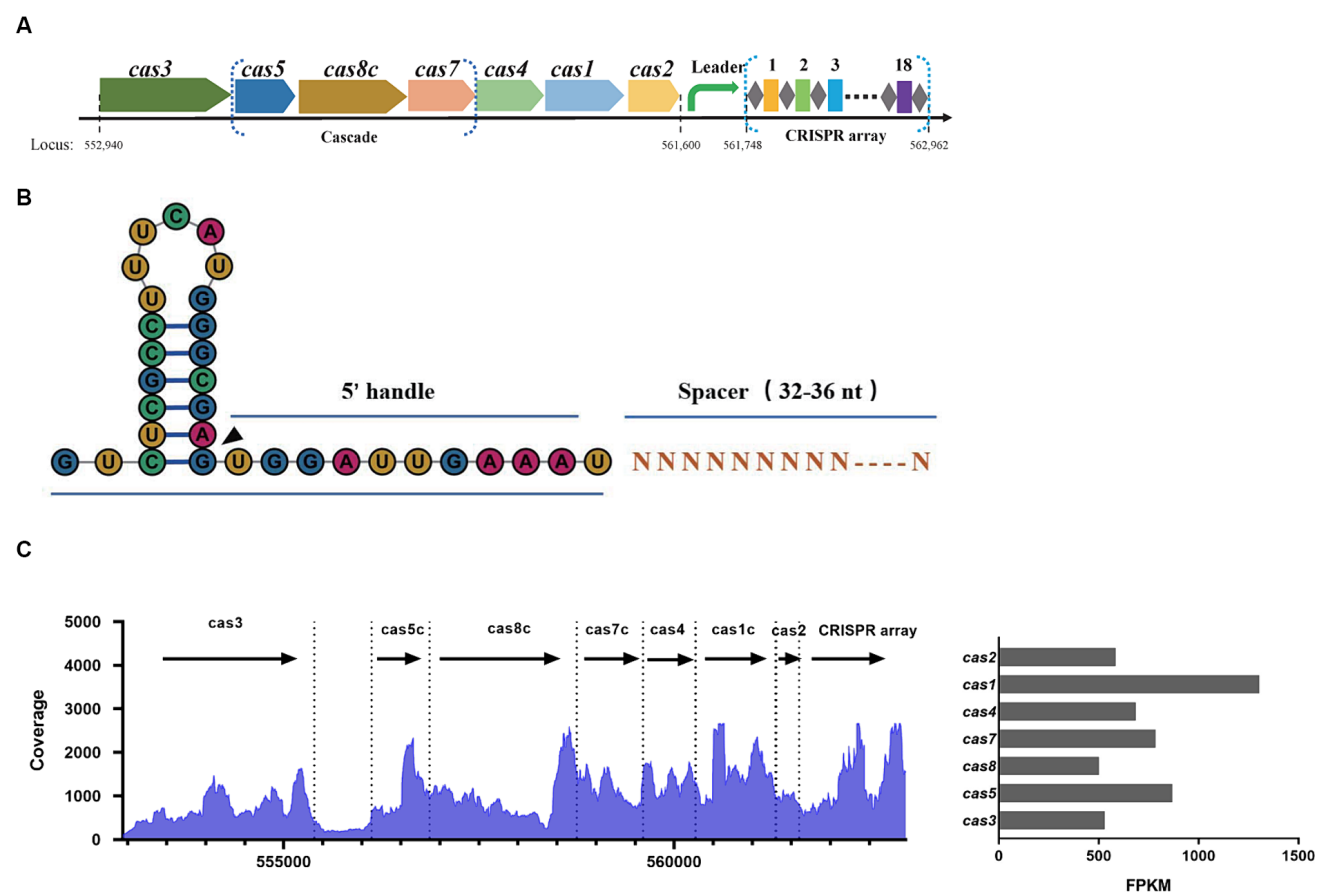
Characterization of the Type I-C CRISPR-Cas system in SEZ ATCC35246. **(A)** Schematic of the Cas locus and the CRISPR array in the genome of SEZ ATCC35246. **(B)** Schematic representation of crRNA. **(C)** Transcriptional profile of the Type I-C CRISPR locus in SEZ, and the bar graph represents the FPKM values of Cas genes.

In the SEZ ATCC35246 strain, a region encoding the Cas protein cluster was identified spanning from position 552,940 to 561,600, which includes Cas3, Cas5, Cas8, Cas7, Cas4, Cas1, and Cas2 (Figure 1A). The subsequent region from 561,748 to 562,962 contained a CRISPR array composed of 18 spacers, with a repeat sequence of 32 nucleotides (5′-GTCTCGCCTTTCATGGGCGAGTGGATTGAAAT-3′). Based on the crRNA structure from other subtype I-C CRISPR systems (Hochstrasser et al., 2016), each mature crRNA in SEZ is predicted to be made up of a 5′ handle (11 nt), a spacer (33–36 nt), and a 3′ stem-loop (21 nt) (Figure 1B). RNA sequencing of SEZ demonstrated the expression of Cas genes and the CRISPR array, indicating the presence of a functional endogenous Type I-C CRISPR-Cas system (Figure 1C).

### Cas3 mutant and CRISPR-deficient strains resulting receded biofilm formation

**Figure 2:**
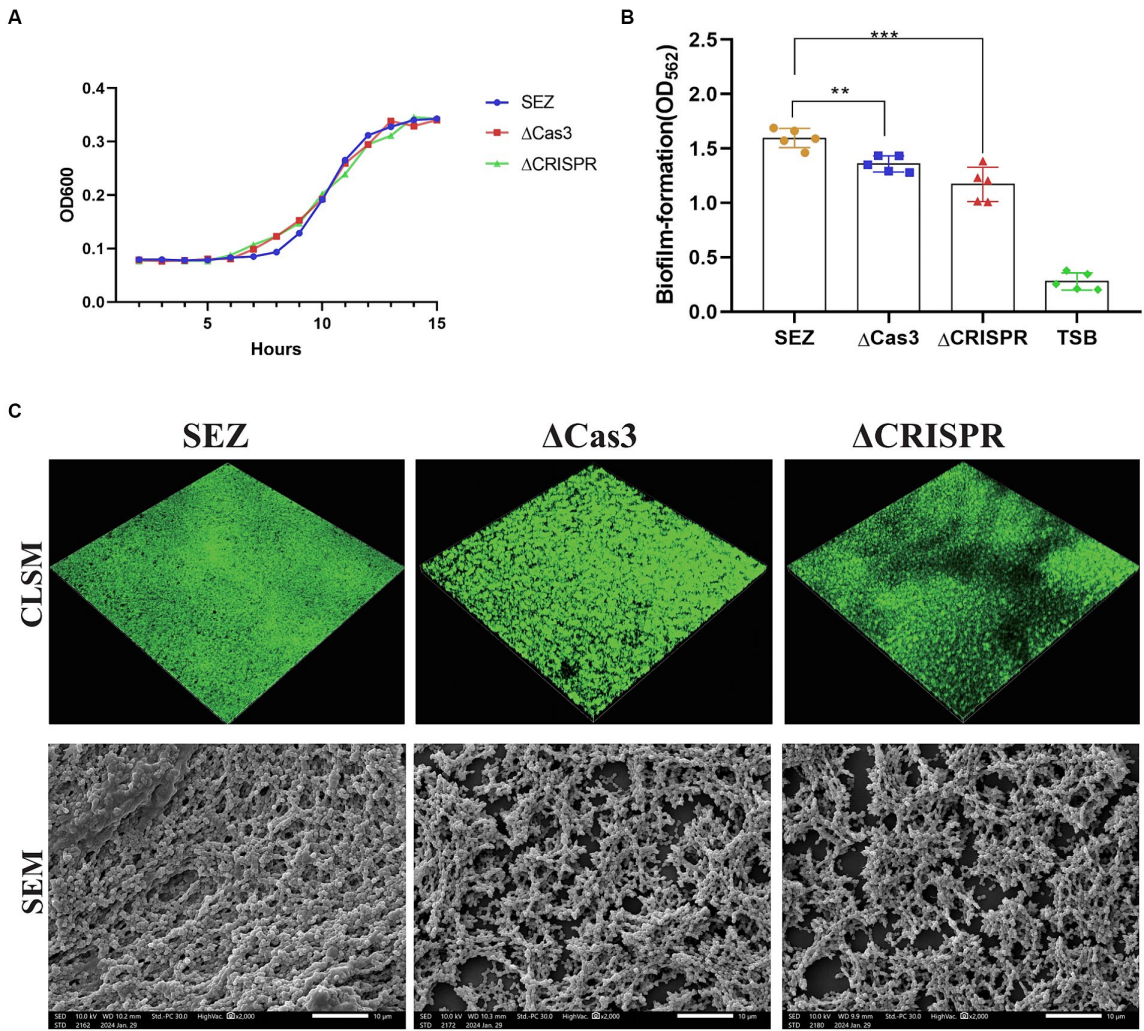
The Type I-C CRISPR-Cas system promotes biofilm formation in SEZ. **(A)** Growth curves of the wild-type SEZ, ΔCas3 (deletion of *cas3* gene), and ΔCRSIPR (CRISPR-deficient strain) strains in THB medium at 37°C, monitored by measuring OD_600_. **(B,C)** Biofilm formation using crystal violet staining, CLSM, and SEM. ^**^p < 0.01, and ^***^p < 0.001.

We successfully constructed two mutant strains, ΔCas3 and ΔCRISPR. Compared to the wild-type SEZ strain, there were no differences observed in bacterial growth (Figure 2A). Previous research has indicated the involvement of the CRISPR-Cas system in bacterial biofilm formation (Cui et al., 2020; Nie et al., 2022). We assessed the biofilm-forming ability of the wild-type SEZ and ΔCas3, ΔCRISPR mutant strains using crystal violet staining. ΔCas3 and ΔCRISPR mutant strains showed significantly reduced biofilm formation compared to the wild-type SEZ strain (Figure 2B).

To further visualize biofilm formation, we employed confocal laser scanning microscopy (CLSM) and scanning electron microscopy (SEM) (Figure 2C). CLSM images revealed that ΔCas3 and ΔCRISPR strains displayed more gaps in biofilm fluorescence, whereas the wild-type SEZ strain exhibited broader and denser fluorescence coverage. Similarly, SEM observations showed that the biofilms of ΔCas3 and ΔCRISPR mutant strains were sparser and structurally looser compared to the wild-type SEZ strain. Collectively, these results confirm that the Type I-C CRISPR-Cas system significantly promotes biofilm formation in SEZ.

### CRISPR-Cas enhances adhesion without altering SEZ morphology or capsule production

**Figure 3:**
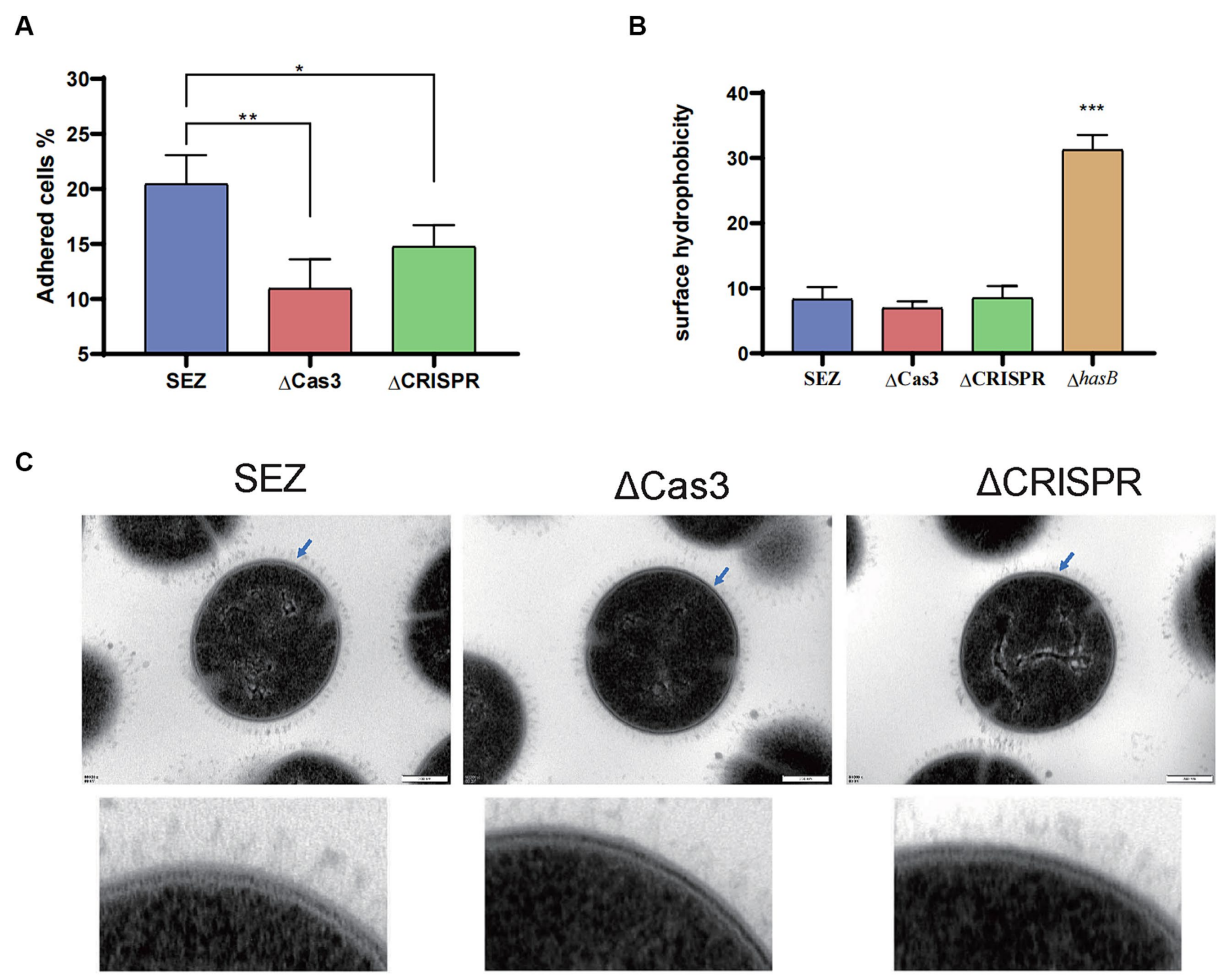
The Type I-C CRISPR-Cas system enhances adhesion without altering SEZ morphology or capsule production **(A)** Adherence of SEZ strains to PK-15 cells, assessed at 1 h post-inoculation. **(B)** Evaluation of surface hydrophobicity of SEZ, ΔCas3, ΔCRISPR and capsular-deficient (Δ*hasB*) strains. **(C)** Transmission electron microscopy images of SEZ strains. ^*^p < 0.05, ^**^p < 0.01, and ^***^p < 0.001.

Then, we compared the adhesion rates of the SEZ strains to PK-15 cells. As shown in Figure 3A, ΔCas3 and ΔCRISPR exhibited significantly reduced adhesion to PK-15 cells. Then hydrophobicity was measured, with no significant differences observed among the wild-type SEZ, ΔCas3 and ΔCRISPR strains (Figure 3B). To further understand whether bacterial morphology was altered, transmission electron microscopy was employed to observe the bacteria. As depicted in Figure 3C, no changes were observed in morphology or capsule among SEZ strains.

### CRISPR-Cas effects transcription of pro-inflammatory cytokines in RAW264.7 macrophages

**Figure 4:**
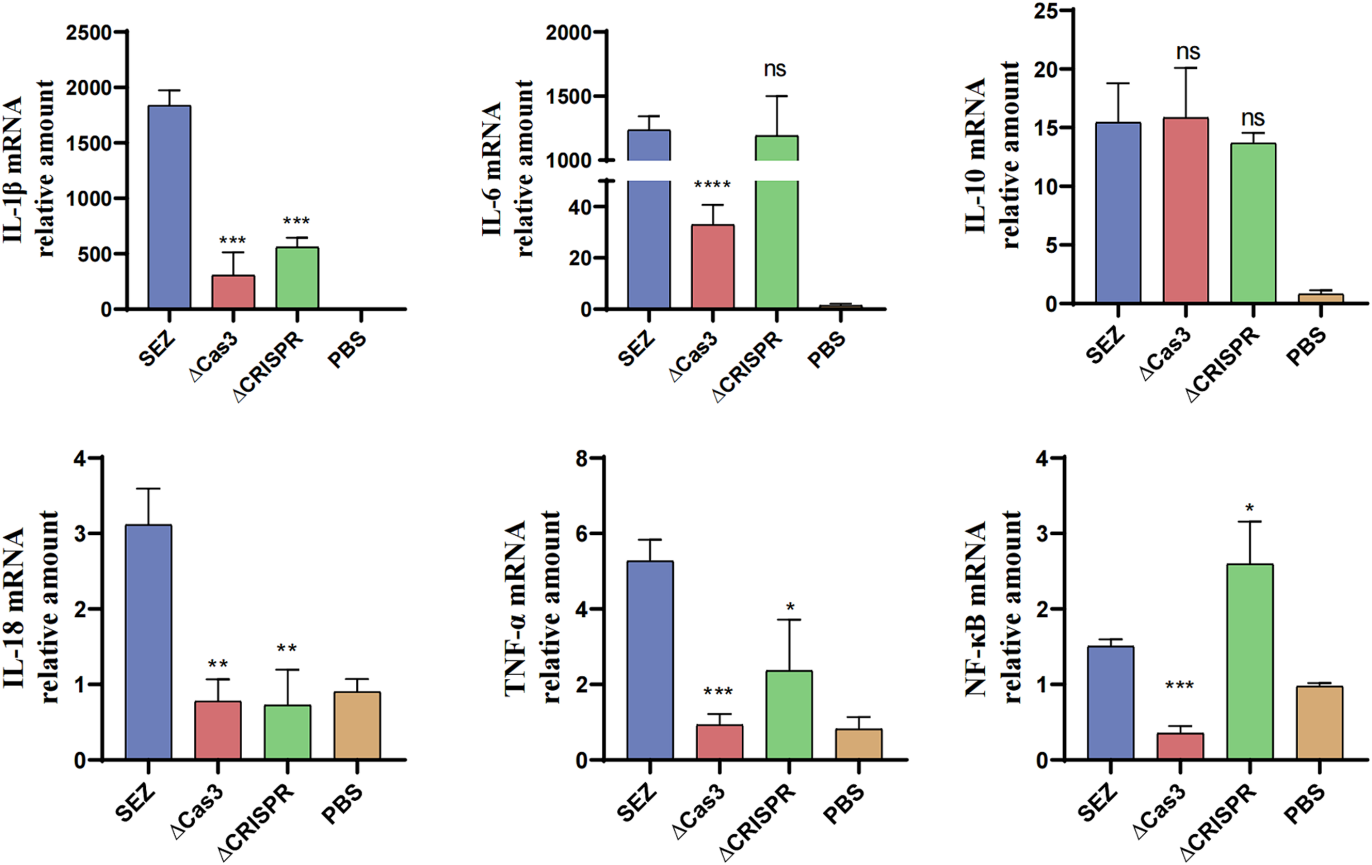
The mRNA expression levels of IL-1β, IL-6, IL-10, IL-18, TNF-α, and the transcription factor NF-κB in RAW264.7 cells were assessed after coculturing with SEZ strains for 12 h at an MOI of 1. The data shown represent average values from three independent experiments. ^*^p < 0.05, ^**^p < 0.01, and ^***^p < 0.001, ns as not significant.

To investigate whether the deletion of Cas3 and CRISPR genes in SEZ affects host immune responses, we conducted infection study using RAW264.7 macrophage cells and measured the transcription levels of several pro-inflammatory cytokines and the transcription factor NF-κB (Figure 4). Compared to wild-type SEZ, the expression levels of IL-1β, IL-18, and TNF-α were significantly reduced in ΔCas3 and ΔCRISPR, while IL-10 transcription remained relatively unchanged. In the ΔCRISPR infection, IL-6 transcription levels showed no significant difference compared to wild-type SEZ, whereas the IL-6 expression induced by ΔCas3 strain was significantly lower than that of wild-type SEZ. NF-κB acts as a key transcription factor in pathogen microbial infections, regulating the expression of multiple inflammatory factors. The mRNA expression of NF-κB in the ΔCas3 was significantly lower than wild-type SEZ, while the opposite was observed in the ΔCRISPR group. This may suggest that ΔCas3 and ΔCRISPR mutant strains affect NF-κB activation through different mechanisms.

### ΔCas3 mutant and CRISPR-deficient strains exhibit reduced pathogenicity in mice

**Figure 5:**
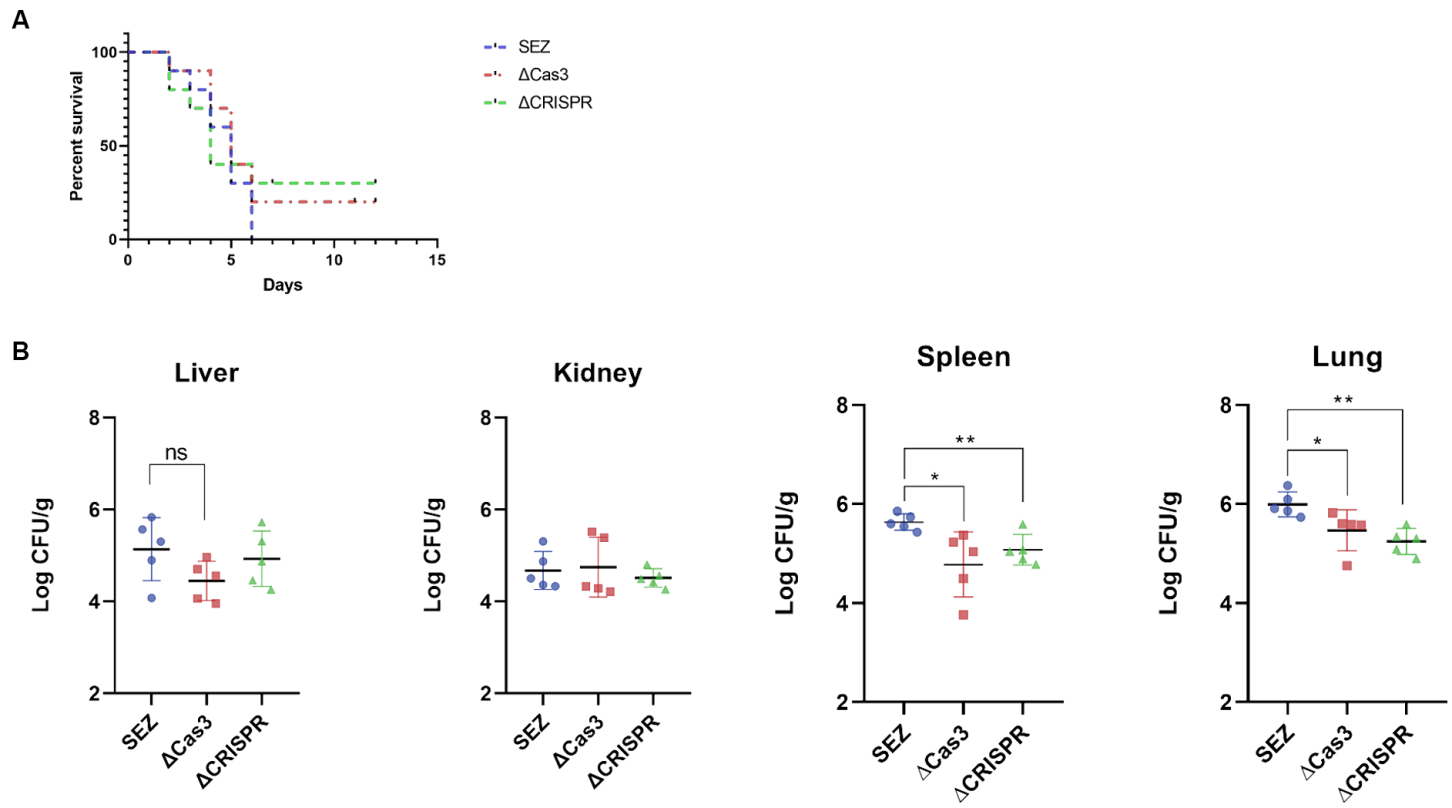
The Type I-C CRISPR-Cas system enhances virulence in mice. 1 × 10^6^ CFU of SES strains were intraperitoneally injected into mice. **(A)** The survival rate of mice was monitored for 12 days post-infection, with *n* = 10 per group. **(B)** Bacterial loads in lungs, livers, kidneys and spleens of the infected mice (*n* = 5 per group) was assessed 24 h post-infection. The bacterial loads were expressed as the number of CFU/g of tissue. ^*^p < 0.05 and ^**^p < 0.01, and ns as not significant.

Previous studies suggest that the CRISPR-Cas system may modulate bacterial pathogenicity in certain species (Shabbir et al., 2018; Solbiati et al., 2020). To investigate the involvement of the Type I-C CRISPR-Cas system in SEZ pathogenicity, we conducted infection experiments using mice. Intraperitoneal injection of 1 × 10^6^ CFU bacteria resulted in 100% mortality within 6 days in the wild-type SEZ infection. In contrast, mortality rates dropped to 70 and 80% for mice infected with the ΔCas3 and ΔCRISPR mutant strains, respectively (Figure 5A). This decrease suggests reduced virulence in these mutants compared to the wild-type SEZ. Furthermore, we noted significantly lower bacterial loads in the spleen and lungs of the mutant-infected mice, with no notable differences in the liver and kidneys (Figure 5B).

### Transcriptomic profile of ΔCas3 and ΔCRISPR mutant strains

**Figure 6:**
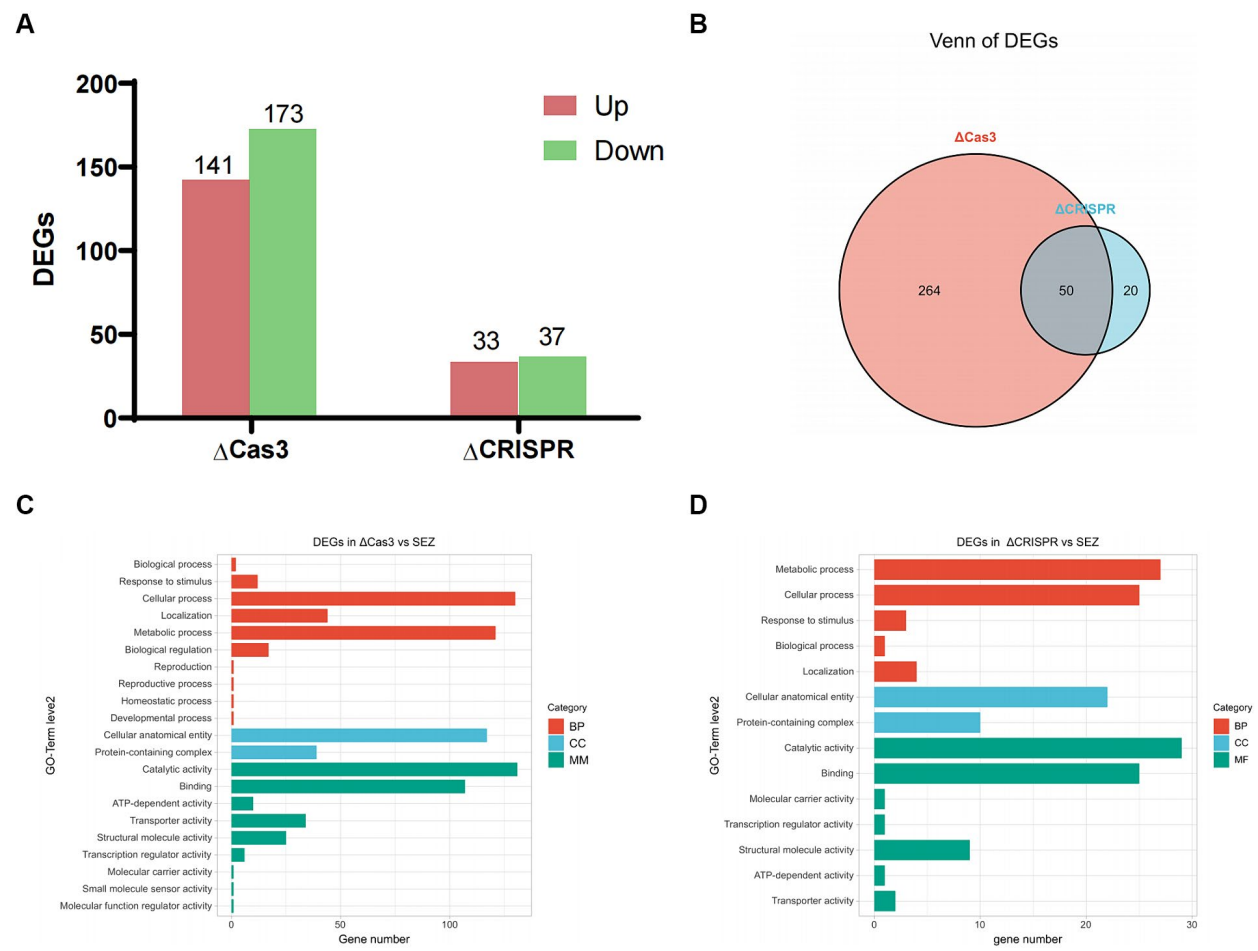
Transcriptomic profile of ΔCas3 or ΔCRISPR compared to the wild-type SEZ strain. **(A)** Number of differentially expressed genes (DEGs) in ΔCas3 and ΔCRISPR mutants. **(B)** Venn diagram analysis of the overlapping differentially expressed genes between ΔCas3 and ΔCRISPR mutants. **(C,D)** GO-term level 2 annotations for the DEGs in ΔCas3 and ΔCRISPR mutants.

To better understand how the CRISPR-Cas system regulates gene expression, we performed RNA-seq analyses on the ΔCas3, ΔCRISPR, and wild-type SEZ strains. Our results indicated significant differences in gene expression profiles: the ΔCas3 strain exhibited 314 significantly differentially expressed genes (DEGs)-141 upregulated and 173 downregulated (Figure 6A). The ΔCRISPR mutant strain displayed 70 DEGs, consisting of 33 upregulated and 37 downregulated genes. Notably, 50 genes were differentially expressed in both ΔCas3 and ΔCRISPR strains, representing over 70% of the differential genes in ΔCRISPR (Figure 6B).

Gene Ontology (GO) level 2 annotation revealed that the DEGs in the ΔCas3 strain primarily relate to cellular processes, metabolic activities, molecular catalysis, binding, and cellular structure and localization (Figure 6C). Similarly, the ΔCRISPR mutant strain has 27 DEGs linked to metabolism, 25 to cellular processes, and 22 to structural components (Figure 6D).

**Figure 7:**
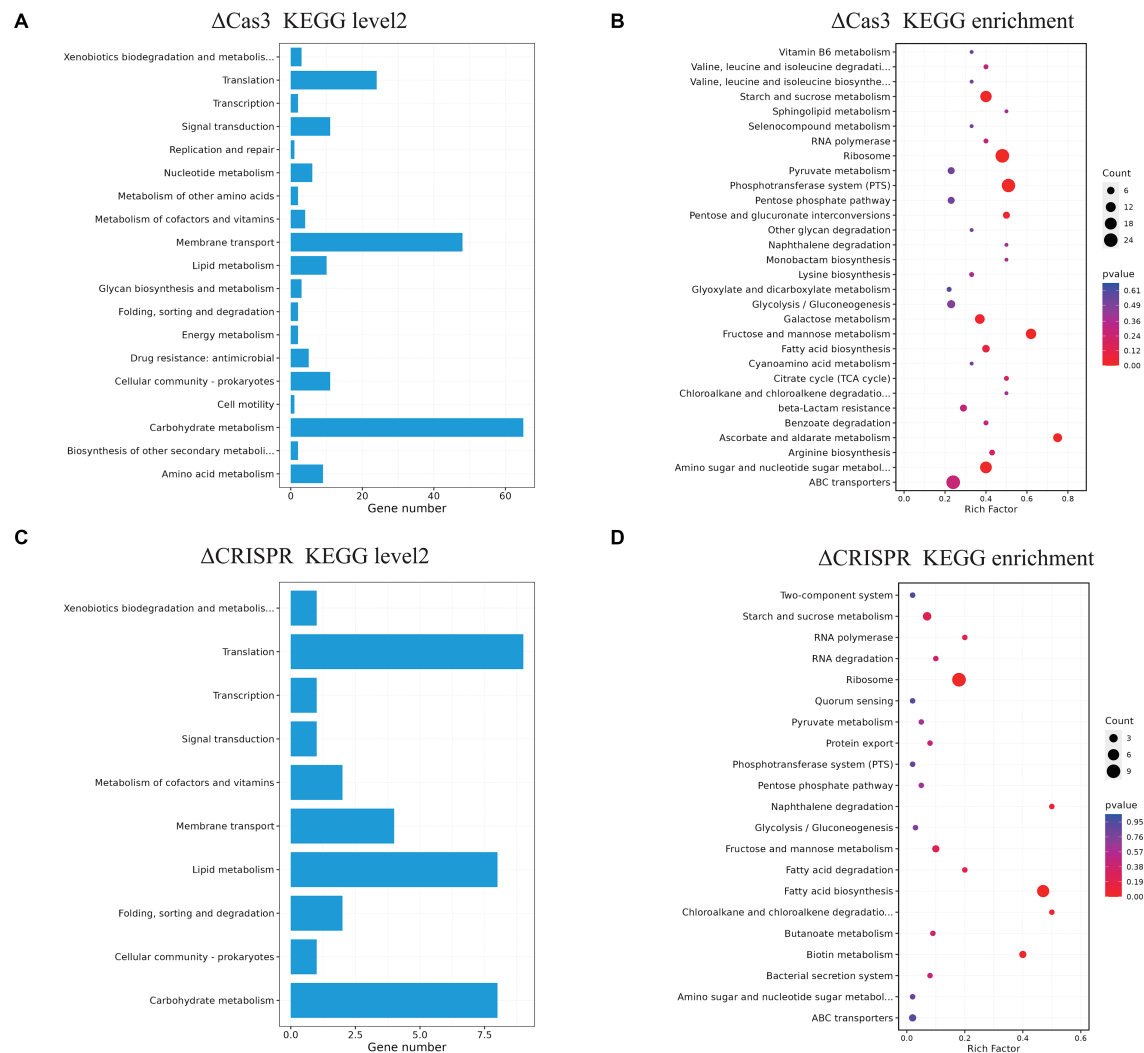
KEGG pathway annotation and enrichment analysis of differentially expressed genes in ΔCas3 and ΔCRISPR mutants. **(A,B)** Represent the level 2 annotation and enrichment for the ΔCas3 mutant strain of DEGs, while **(C,D)** show the KEGG analysis for the DEGs in the ΔCRISPR mutant. The size of each point indicates the number of genes involved in the pathway.

The ΔCas3 mutant strain shows a large number of DEGs, indicating its regulatory characteristics are more complex. To further understand the functional classification of these genes, we annotated the DEGs of ΔCas3 and ΔCRISPR using the KEGG database (Figures 7A–D). The ΔCas3 annotation reveals a substantial number of genes mapped to carbohydrate metabolism (65 genes) and a significant representation in membrane transport (involving 48 genes). Additional associations include amino acid metabolism, lipid metabolism, quorum sensing, signal transduction, and drug resistance. Enrichment analysis highlighted significant activity in primary metabolic processes, especially pathways related to energy metabolism such as carbohydrate, amino sugar, and nucleotide sugar metabolism, as well as protein biosynthesis, including ribosomal component expression. The ΔCRISPR mutant also showed similar patterns in gene expression, primarily involving protein translation, lipid and carbohydrate metabolism, and membrane transport processes mediated by ABC transporters.

**Figure 8:**
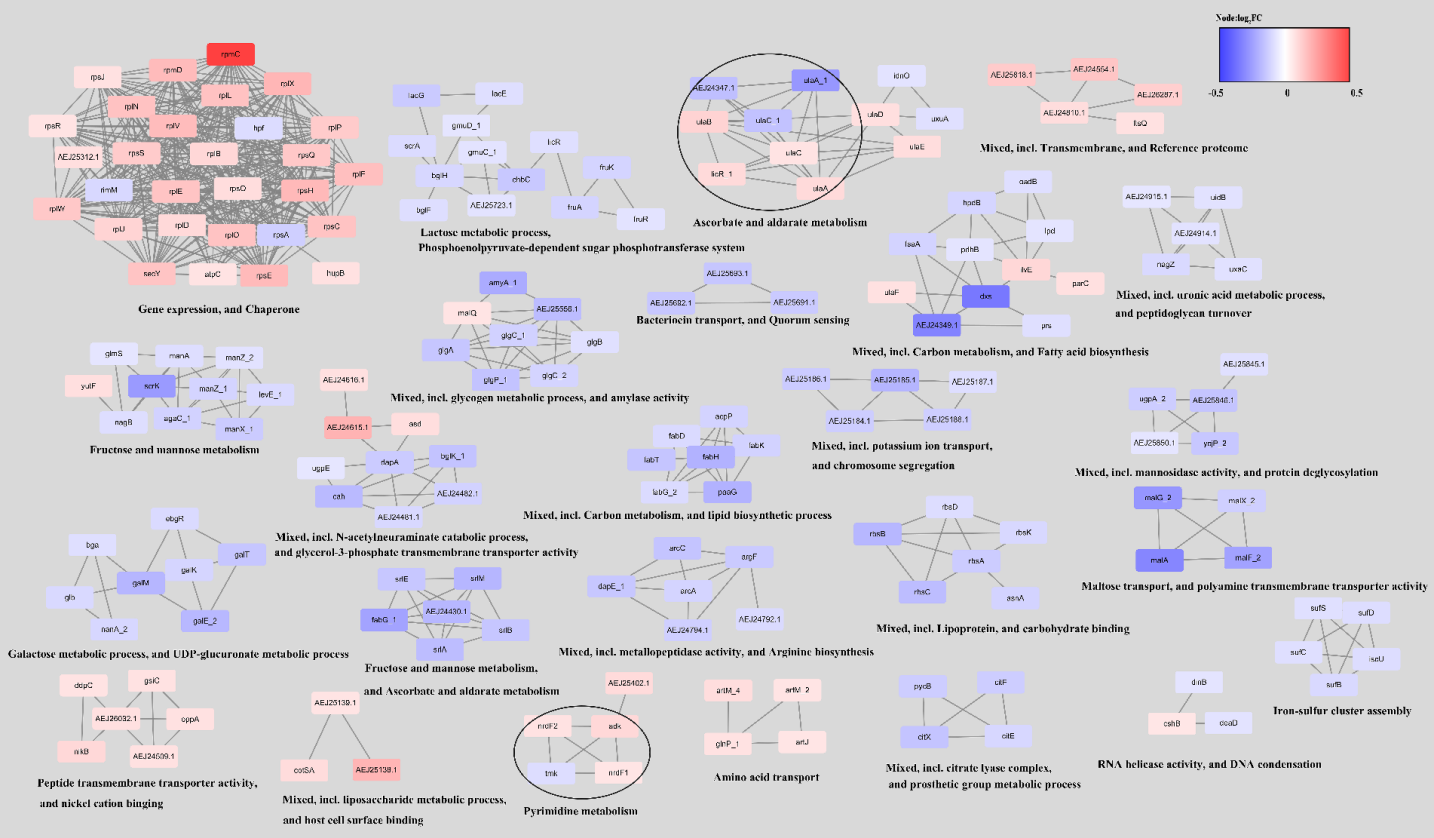
Network of differentially expressed genes in the ΔCas3 mutant strain. To construct this network, the genes were queried in the STRING database for the SEZ ATCC35246 using a confidence (score) cutoff of 0.7 and allowing a maximum of 10 additional interactors. The network was then clustered using the MCL Cluster algorithm, which is implemented in the Cytoscape plugin clusterMaker, to identify clusters and functional interactions. For each cluster, functional enrichment was performed using the stringApp to obtain enriched terms.

Next, utilizing the String database, we constructed a network analysis of the DEGs in the ΔCas3 mutant strain, as illustrated in Figure 8. The upregulated genes are primarily involved in gene expression regulation, protein folding, transmembrane transport, and host interactions. Conversely, the downregulated genes are mostly associated with primary metabolic processes, including the metabolism of carbohydrates, lipids, and amino acids, as well as with quorum sensing systems and transport processes. In summary, the observed changes in ΔCas3 mutants, regarding metabolism regulation, stress response, interaction with the host, and intracellular communication, suggest significant impacts on their physiological state and interaction with the host.

## Discussion

SEZ is an emerging pathogen of pigs in the Western Hemisphere, and disease is particularly associated with ST-194 (Costa et al., 2022). Several virulence factors of SEZ have been characterized, including the capsule composed of hyaluronic acid (Wei et al., 2012), the ability to form biofilms (Yi et al., 2014), and the M-like protein SzM, which binds host proteins such as fibrinogen and IgG (Bergmann et al., 2019; Song et al., 2022). The expression of the hyaluronic acid capsule and SzM is critical for the survival and proliferation of SEZ in the bloodstream. Additionally, the recently discovered BifA protein, a Fic domain-containing protein, has been shown to disrupt the integrity of the blood-brain barrier by activating moesin in endothelial cells (Ma et al., 2019; Pan et al., 2022). Here, we found that the SEZ outbreak isolates in both the United States and China carried the Type I-C CRISPR-Cas system. This observation leads us to speculate that the Type I-C CRISPR-Cas system may play a role in regulating SEZ virulence. Furthermore, we have characterized the endogenous CRISPR-Cas system in the SEZ ATCC3246 strain, demonstrating its involvement in regular transcriptional activities. This highlights the system’s completeness and underscores its potential functionality.

Biofilms provide pathogenic bacteria with advantages for their survival against diverse stresses and enhance their pathogenicity in host environments (Solano et al., 2014). In present study, we noted a reduction in biofilm formation capabilities in the ΔCas3 and ΔCRISPR mutant strains. Previous studies have demonstrated that the deletion of Cas3 in both *Salmonella* and *Streptococcus mutans* leads to decreased biofilm formation (Tang et al., 2019; Cui et al., 2020). Moreover, research on *Streptococcus agalactiae* has indicated that CRISPR contributes to biofilm formation (Nie et al., 2022). These studies collectively support our findings, suggesting CRISPR-Cas systems play a role in biofilm formation.

The ability of SEZ to invade mucous membranes, migrate into different tissues, and penetrate the blood–brain barrier is essential for its transition from infection to invasive disease (Xu et al., 2016). Cell adhesion is a crucial step in the pathogenesis of SEZ, and we observed that the deletion of the Cas3 and CRISPR significantly inhibited SEZ adhesion to PK-15 cells. Referring to previous studies, it has been found that CRISPR in *Streptococcus agalactiae* affects adhesion by inhibiting the production of capsule polysaccharides (Nie et al., 2022). Interestingly, here, we did not observe any changes in the morphology of SEZ cells or in polysaccharide production. This indicates that CRISPR-Cas does not directly affect SEZ cell morphology or capsule production. The outer layer of the cell wall of SEZ is enveloped by a layer of capsule polysaccharide composed of hyaluronic acid, which plays a crucial role in the process of bacterial invasion into the host organism by protecting the bacteria from being engulfed by various immune system phagocytic cells (Xu et al., 2016). In fact, the capsule production is regulated by the has operon, which involves five genes (*hasA, hasB, hasC, glmU, and pgi*) (Blank et al., 2008).

The deletion of Cas3 or CRISPR changed the pro-inflammatory response of RAW264.7 cells. We observed that the expression levels of IL-1β, IL-18, and TNF-α were significantly reduced in the SEZ mutants. Unexpectedly, this does not align with the previously thought mechanism that the CRISPR-Cas system controls virulence through the suppression of immune responses. The CRISPR-Cas systems of *Pseudomonas* PA14 have been shown to modify the levels of lasR mRNA, thereby influencing the immune response of macrophages (Li et al., 2016). This is due to PA14 evading recognition and clearance by host immune cells mediated by TLR4 responses, resulting in reduced inflammatory reactions. The Type II CRISPR-Cas systems of *Francisella novicida* are implicated in influencing virulence, leading to altered host defense by decreasing the expression of bacterial lipoprotein (Sampson et al., 2013). The deletion of Cas3 had a significant effect on the immune response of THP-1 cells to *Porphyromonas gingivalis*, with IL-1β and TNF-α genes being upregulated in cells infected with the mutant (Solbiati et al., 2020). Our study, however, was limited to a quantitative analysis of mRNA levels in infected cells, pointing to the need for further research to fully understand these dynamics. Notably, recent work on the Type III-A CRISPR system in *Mycobacterium tuberculosis* highlights its direct impact on host immune processes, suggesting that CRISPR-Cas proteins could act as virulence factors by modulating host immune and inflammatory pathways, potentially exacerbating tissue damage (Jiao et al., 2021; Wu et al., 2022). This evolving understanding calls for a reevaluation of the roles of CRISPR-Cas systems, focusing on their interaction with host immunity. Our findings underscore the need for further investigation into the diverse roles CRISPR-Cas systems may play in bacterial pathogenesis during host infection.

Regardless, the CRISPR-Cas system is significantly linked to bacterial pathogenicity. For example, this system may increase invasive capabilities, with its disruption potentially reducing virulence in *Salmonella enterica* (Cui et al., 2020). In *Streptococcus mutans*, Cas3 deletion significantly downregulates virulence genes like *vicR*, *gtfC*, *smu0630*, and *comDE* (Tang et al., 2019). Our study further corroborates this association. Here, in mice infection assay, the absence of Cas3 or CRISPR improved survival rates, with a notable reduction in bacterial load in the spleen and lungs compared to wild-type SEZ. These findings collectively suggest that the CRISPR-Cas system likely plays a role in regulating SEZ virulence and influences bacterial proliferation and dissemination in specific organs.

RNA-seq analysis was employed to investigate variations in gene regulation among two SEZ mutant strains grown under planktonic conditions during the logarithmic phase of growth. We observed notable differences in the number of differentially expressed genes between ΔCas3 and ΔCRISPR. Specifically, ΔCas3 displayed a more extensive array of differentially expressed genes, suggesting broader biological changes. Crucially, we identified 50 overlapping differentially expressed genes between the two strains, representing over 70% of the differential genes in ΔCRISPR. This finding indicates parallel trends in gene expression modifications, which may reflect a uniform biological response to CRISPR-Cas system alterations or consistent traits within certain biological pathways.

GO and KEGG pathway analyses showed similar patterns of enrichment among differentially expressed genes in the ΔCas3 and ΔCRISPR strains, particularly in basic metabolism, such as fatty acid and carbohydrate metabolism, and significant changes in signal transduction and membrane transport proteins like the ABC transport system and the phosphotransferase system, as well as quorum sensing. While some studies propose a connection between the CRISPR-Cas system and quorum sensing systems (Patterson et al., 2016; Semenova and Severinov, 2016), the latter serves as a crucial mechanism governing signal delivery in microorganisms and plays a vital role in gene expression regulation (Azimi et al., 2020). Our previous studies indicated that the LuxS quorum sensing system in SEZ might negatively regulate the transcription of the CRISPR-Cas system (Xie et al., 2024). However, in the RNA-seq data of ΔCas3 and ΔCRISPR, we did not observe alterations in LuxS expression. Actually, SEZ also possesses the autoinducer peptide-type quorum sensing system (Xie et al., 2019), with significant transcriptional changes observed in quorum sensing related genes and ABC transport genes in both ΔCas3 and ΔCRISPR mutants, suggesting a complex interplay between these two systems. Given the widespread presence of these systems in bacteria, their interconnected roles or potential regulatory impacts merit further investigation.

In both ΔCas3 and ΔCRISPR mutant strains, we observed an enrichment of fatty acid metabolism. Lipids, which are crucial for maintaining the integrity of the bacterial cell wall, play a significant role in this process (MacDermott-Opeskin et al., 2022). Yang et al. (2021) discovered that deleting the Type III-A CRISPR-Cas System in *Mycobacterium* affects fatty acid metabolism, increases sensitivity to hydrogen peroxide, and adversely affects cell envelope integrity. Although transmission electron microscopy revealed no significant alterations in the morphology or capsule structure of the ΔCas3 and ΔCRISPR strains, we speculate that these changes in lipid synthesis might influence their capabilities for cell adhesion and biofilm formation in SEZ.

In summary, this study offers a relatively comprehensive characterization of the role of the CRISPR-Cas system in SEZ. The CRISPR-Cas system is involved in the formation of biofilms and affects cell adhesion in SEZ, yet it does not impact bacterial morphology or capsule production. The deletion of Cas3 or CRISPR affects the transcription of certain pro-inflammatory factors in RAW264.7 cells. Additionally, we demonstrated that this system enhances the virulence of SEZ in mice. The transcriptomic profile of the ΔCas3 and ΔCRISPR mutant strains revealed the important role of the Type I-C CRISPR-Cas system in SEZ biology. These findings enhance our understanding of its complex functions within SEZ and provide crucial insights for developing new anti-infection treatment strategies.

## Data availability statement

The datasets presented in this study can be found in online repositories. The names of the repository/repositories and accession number(s) can be found in the article/Supplementary material.

## Ethics statement

All operations in this study involving animal experiments were handled according to the Ethics Committee at Northwest A&F University (approval number DY2022009). The study was conducted in accordance with the local legislation and institutional requirements. The study was conducted in accordance with the local legislation and institutional requirements.

## Author contributions

HX: Conceptualization, Data curation, Formal analysis, Investigation, Methodology, Software, Writing – original draft, Writing – review & editing. RZ: Data curation, Formal analysis, Software, Writing – review & editing. ZL: Writing – review & editing, Data curation, Investigation. RG: Methodology, Validation, Writing – review & editing. JL: Investigation, Methodology, Writing – review & editing. QF: Resources, Supervision, Writing – review & editing. XW: Supervision, Writing – review & editing. YZ: Funding acquisition, Resources, Writing – review & editing.
