# Supplementary material for: Endogenous Type I-C CRISPR-Cas system of Streptococcus equi subsp. zooepidemicus promotes biofilm formation and pathogenicity
Source: Front Microbiol. 2024 May 22;15:1417993. doi: 10.3389/fmicb.2024.1417993 (PMC11150851; doi:10.3389/fmicb.2024.1417993)
Supplement: Supplementary file 1 [file Table_1.DOCX]

| Strain | Location | Isolate  time | Host | System type | Number of repeats |
| --- | --- | --- | --- | --- | --- |
| ATCC 35246 | China, Sichuan | 1975 | porcine | type I-C | 18 |
| CY | China, Nanjing | 1998 | porcine | type I-C | 18 |
| NVSLTN-TC1 | USA, Tennessee | 2019 | porcine | type I-C | 19 |
| NVSLTN-TB1 | USA, Tennessee | 2019 | porcine | type I-C | 19 |
| TN-714097 | USA, Ohio | 2019 | porcine | type I-C | 19 |
| OH-71905 | USA, Ohio | 2019 | porcine | type I-C | 19 |
| IN-6992 | USA, Indiana | 2021 | porcine | type I-C | 18 |

The list of CRISPR-Cas Systems in SEZ Isolates from Swine Outbreaks in China and the United States
