# Supplementary material for: Endogenous Type I-C CRISPR-Cas system of Streptococcus equi subsp. zooepidemicus promotes biofilm formation and pathogenicity
Source: Front Microbiol. 2024 May 22;15:1417993. doi: 10.3389/fmicb.2024.1417993 (PMC11150851; doi:10.3389/fmicb.2024.1417993)
Supplement: Supplementary file 2 [file Table_2.DOCX]

| **Table 2** | | |
| --- | --- | --- |
| **Bacterial strains and plasmids used in this study.** | | |
| **Plasmid or strain** | **Relevant characteristics** | **References; character** |
| **PASMIDS** |  |  |
| pSET4s | *S. suis* thermosensitive suicide vector; Spc^R^ | (Takamatsu et al., 2001b) |
| pMD19-T | Clone vector | Takara |
| **STRAINS** |  |  |
| SZE | S. *equi* *subsp.* *zooepidimicus* ATCC35246, wild-type strain | (Ma et al., 2011) |
| ΔCas3 | Deletion of the *cas3* gene in SEZ | this study |
| ΔCRISPR | A CRISPR-deficient strain | this study |
| Δ*hasB* | The SEZ capsule-deficient strain | (Wei et al., 2012) |
| Spc^R^, spectinomycin resistant | | |
| **Primers used in this study.** | | |
| **Primer** | **Sequence (5′–3′)** | **character** |
| **Construction of the Cas3 and CRISRP mutant strains** | | |
| Cas3L1 | GGATCCATTAGTGATGATGACTGGCATAG | BamHI, 627bp |
| Cas3L2 | GCAGGCTCCAGATCTGTTTTGCCTTGATAGTGAGCCAATATCATATATGA |  |
| Cas3R1 | AAGGCAAAACAGATCTGGAGCCTGCCCTTCAGGAGCATTATTATCATCAA | EcoRI, 549bp |
| Cas3R2 | GAATTCATATTTGTCCTGCAAAGCTACTT |  |
| CrisprL1 | GGATCCTACTGATGATGGTATTGGTTAC | BamHI,  432bp |
| CrisprL2 | CAGTTTATAGAGAAATGATATAGGCTAGCTACTAACATATTCAGCTATAA |  |
| CrisprR1 | GCCTATATCATTTCTCTATAAACTGCATTAATCAAGTTGATTTAAGCT | EcoRI,  523bp |
| CrisprR2 | GAATTCGAGGATTACACCACCTTACCTGAAA |  |
| **Real-time PCR in RAW264.7 cells** | | |
| IL-1β-F | GCAACTGTTCCTGAACTCAACT | NCBI Reference Sequence: NM_008361.4 |
| IL-1β-R | ATCTTTTGGGGTCCGTCAACT |  |
| IL-6-F | TAGTCCTTCCTACCCCAATTTCC | NCBI Reference Sequence: NM_031168.2 |
| IL-6-R | TTGGTCCTTAGCCACTCCTTC |  |
| TNF-α-F | CCTGTAGCCCACGTCGTAG | NCBI Reference Sequence: NM_013693.3 |
| TNF-α-R | GGGAGTAGACAAGGTACAACCC |  |
| GAPDH-F | TGGCCTTCCGTGTTCCTAC | NCBI Reference Sequence: NM_008084.3 |
| GAPDH-R | GAGTTGCTGTTGAAGTCGCA |  |
| IL-18-F | GACTCTTGCGTCAACTTCAAGG | NCBI Reference Sequence: NM_008360.2 |
| IL-18-R | CAGGCTGTCTTTTGTCAACGA |  |
| NF-κB-F | AGGCTTCTGGGCCTTATGTG | NCBI Reference Sequence: NM_008689.3 |
| NF-κB-R | TGCTTCTCTCGCCAGGAATAC |  |
| IL-10-F | GCTCTTACTGACTGGCATGAG | NCBI Reference Sequence: NM_010548.2 |
| IL-10-R | CGCAGCTCTAGGAGCATGTG |  |

Ma Z, Geng J, Zhang H, Yu H, Yi L, Lei M, Lu C P, Fan H J, Hu S (2011). Complete genome sequence of *Streptococcus equi* *subsp.* *zooepidemicus* strain ATCC 35246. J Bacteriol, 193(19): 5583-5584

Takamatsu D, Osaki M, Sekizaki T (2001a). Construction and characterization of *Streptococcus suis*-*Escherichia coli* shuttle cloning vectors. Plasmid, 45(2): 101-113

Wei Z, Fu Q, Chen Y, Cong P, Xiao S, Mo D, He Z, Liu X. The capsule of *Streptococcus equi* ssp. *zooepidemicus* is a target for attenuation in vaccine development. Vaccine. 2012 Jun 29;30(31):4670-5.

Wei Z, Fu Q, Chen Y, Cong P, Xiao S, Mo D, He Z, Liu X (2012). The capsule of *Streptococcus equi* ssp*. zooepidemicus* is a target for attenuation in vaccine development. Vaccine, 30(31): 4670-4675
